# Quality and accuracy of online nutrition-related information: a systematic review of content analysis studies

**DOI:** 10.1017/S1368980023000873

**Published:** 2023-07

**Authors:** Emily Denniss, Rebecca Lindberg, Sarah A McNaughton

**Affiliations:** Institute for Physical Activity and Nutrition (IPAN), School of Exercise and Nutrition Sciences, Deakin University, 221 Burwood Highway, Burwood, VIC 3125, Australia

**Keywords:** Nutrition information, Online nutrition environment, Information quality, Information accuracy, Systematic review

## Abstract

**Objective:**

This systematic review aimed to summarise the level of quality and accuracy of nutrition-related information on websites and social media and determine if quality and accuracy varied between websites and social media or publishers of information.

**Design:**

This systematic review was registered with PROSPERO (CRD42021224277). CINAHL, MEDLINE, Embase, Global Health and Academic Search Complete were systematically searched on 15 January 2021 to identify content analysis studies, published in English after 1989, that evaluated the quality and/or accuracy of nutrition-related information published on websites or social media. A coding framework was used to classify studies’ findings about information quality and/or accuracy as poor, good, moderate or varied. The Academy of Nutrition and Dietetics Quality Criteria Checklist was used to assess the risk of bias.

**Setting:**

N/A.

**Participants:**

N/A.

**Results:**

From 10 482 articles retrieved, sixty-four were included. Most studies evaluated information from websites (*n* 53, 82·8 %). Similar numbers of studies assessed quality (*n* 41, 64·1 %) and accuracy (*n* 47, 73·4 %). Almost half of the studies reported that quality (*n* 20, 48·8 %) or accuracy (*n* 23, 48·9 %) was low. Quality and accuracy of information were similar on social media and websites, however, varied between information publishers. High risk of bias in sample selection and quality or accuracy evaluations was a common limitation.

**Conclusion:**

Online nutrition-related information is often inaccurate and of low quality. Consumers seeking information online are at risk of being misinformed. More action is needed to improve the public’s eHealth and media literacy and the reliability of online nutrition-related information.

Box 1. Key definitions

Quality: the reliability of information, compared against a set of defined criteria, which usually includes assessment of financial disclosures, citing of references, transparency and provision of balanced and unbiased information^(10)^.

Accuracy: the factual correctness of information, typically in comparison to scientific literature or guidelines published by an authoritative group.

Publisher: the entity that has published information on a website or social media, for example, government or commercial organisation.

Dietary patterns have a significant influence on human health, and poor diet quality is the leading preventable risk factor contributing to the global burden of non-communicable disease^(1)^. Dietary behaviours are complex and have many influences that extend beyond physiological cues such as hunger and taste preferences^(2)^. Social and built nutrition environments also exert an influence on dietary behaviours, including nutrition information environments, which encompass the media and advertising^(3)^. Online environments are virtual, computer-based environments that are connected by the Internet, including websites and social media, and are now a prominent part of the media, with 60 % of the global population having Internet access and higher rates observed in high-income countries^(4)^. The WHO has outlined that such online environments can influence dietary behaviours through the provision of services and information^(5)^.

In recent years, the prevalence and spread of health misinformation in online platforms have become a significant problem. In 2013, the World Economic Forum marked digital misinformation as one of the most dangerous trends of the era^(6)^. Since then, the spread of health misinformation online has contributed to vaccine hesitancy, the ‘anti-vax’ movement and likely contributed to the spread of COVID-19^(7,8)^. Internet and social media users can instantaneously publish information on any topic, regardless of their expertise or qualifications. Consequently, consumers are presented with an abundance of online information of variable quality and veracity^(9,10)^. Furthermore, it has been identified that consumers typically have low levels of media literacy and critical evaluation skills^(11,12)^. These factors have led to a scenario in which time-poor consumers are inundated with online information that they are unable to adequately scrutinise^(13)^.

Dietitians, public health nutritionists and organisations have raised concerns about the potential for nutrition-related misinformation to cause harm^(5,14)^ and as a barrier to healthy eating behaviours^(15)^. Consumers are increasingly relying on the Internet and social media for nutrition-related information^(16–20)^, which puts them at risk of being misinformed. Further, the public’s trust in nutrition science and authoritative voices in the field has been eroded^(21,22)^. Numerous factors have contributed to the erosion of trust, including scientific uncertainty^(23)^, failure to disclose conflicts of interest^(21,22)^, insufficient context in nutrition communication and contradictory messaging about nutrition issues^(14)^. Exposure to nutrition information that lacks context or seems contradictory can lead to confusion and backlash among consumers^(24,25)^. In turn, consumers are less likely to accept nutrition information from authoritative experts and may rely on information from less credible and qualified sources, further increasing their risk of being misinformed^(24,25)^.

The quality and accuracy of health information on websites and social media have been extensively researched. Numerous systematic reviews have summarised the literature about the quality or accuracy of health information on the Internet and social media, to provide a more comprehensive overview of the information landscape^(10,26–28)^. These reviews are able to capture large amounts of data about the quality or accuracy of online health information, which is not feasible in a single study, due to the time-intensive process of quality and accuracy assessments, the plethora of information online and the continuous cycle of information being published, updated and deleted. However, to date, no systematic reviews have been conducted that summarise the quality or accuracy of online information specific to nutrition. Therefore, the aims of the current review were to systematically search the literature in order to: (1) summarise the level of quality and accuracy of nutrition-related information in online environments and (2) determine if nutrition-related information’s quality and accuracy varied between websites and social media or different publishers of information.

## Methods

The protocol for this systematic review was registered in PROSPERO: CRD42021224277 (https://www.crd.york.ac.uk/prospero/display_record.php?RecordID=224277) in January 2021 and followed the Preferred Reporting Items for Systematic Reviews and Meta-Analysis (PRISMA)^(29)^ and the PRISMA literature search extension (PRISMA-S) protocols^(30)^. The PRISMA checklist is included in online Supplementary Table 1.

### Inclusion and exclusion criteria

Peer-reviewed content analysis studies published in English after January 1989 that evaluated the quality and/or accuracy of nutrition-related information in online environments (websites or social media) were eligible for inclusion. For the purposes of this review, nutrition-related information was defined as information regarding healthy eating, dietary patterns, nutrients, nutritional requirements, nutritional composition of foods, nutritional supplements, health outcomes associated with foods and dietary patterns, food safety, food ethics and cooking. This definition was developed to incorporate key components of food literacy as defined by Vigden *et al*.^(31)^ The year 1989 was selected because it is the year the world wide web became available^(32)^. Studies that evaluated information from only one website or information intended for health professionals or experts were excluded. Conference abstracts, theses, unpublished works, editorials, perspectives, commentaries, systematic reviews and original research that used methods other than content analysis were excluded. Studies that focused specifically on online advertising were also excluded because food and nutrition-related advertising has been extensively researched and is beyond the scope of this review.

### Search strategy

CINAHL (EBSCOhost), MEDLINE Complete (EBSCOhost), Embase (Ovid), Global Health (EBSCOhost) and Academic Search Complete (EBSCOhost) were systematically searched on 15 January 2021. Each database was searched individually. Study titles and abstracts were searched, and the search strategy included search terms related to four concepts: nutrition; AND online environments; AND quality/accuracy; AND information. Terminology was altered to include subject headings relevant to the database being searched. The databases and search terms used were decided upon after extensive pilot testing and consultation with the health librarian. Searches were limited to peer-reviewed journals and articles published after January 1989. To ensure that no relevant articles were missed, backwards and forwards searching of included articles was performed through hand searching of reference lists and Scopus searches of citing articles. Scopus searches were performed on 25 October 2021. See online Supplementary File 1 for further details of search strategy.

### Screening

Results from database searches were downloaded and saved in an Endnote library (version X9), which was imported to Covidence software (Veritas Health Innovation). Duplicates were automatically removed during the import, and title and abstract screening was conducted in Covidence. Two researchers (ED and SM/RL) independently screened each article to determine its eligibility. Title and abstract screening disagreements were resolved by the researcher who did not initially screen the disagreed upon reference. Full-text articles were also independently reviewed by two authors (ED and SM/RL). Disagreements were discussed among all authors until consensus was reached.

### Data extraction

A data extraction template was developed and was informed by a previous scoping of the literature and the systematic review aims. One author (ED) independently extracted data from all included references in Microsoft Excel (version 2108). If an included study contained components that were unclear or difficult to extract, the paper was circulated to all authors who met to discuss until the issue was resolved. The following data were extracted: study details (year of publication, country of origin, title, corresponding author’s contact details, aim, online environment investigated, nutrition-related topic of interest), methods (search strategy, inclusion and exclusion criteria, method of quality and/or accuracy evaluation, method of assessing inter-rater reliability), results (sample size, findings about information quality and/or accuracy and inter-rater reliability) and conflicts of interest. If a study focused on a broad health topic, only information relevant to nutrition was extracted.

### Data synthesis

To assist in the interpretation of quality and accuracy findings, a classification framework developed for previous systematic reviews on health information quality was adapted^(10,26)^. Quality or accuracy was coded as: (1) poor, if the authors’ overall tone about the quality or accuracy of the information was cautious or unfavourable; (2) good, if authors spoke positively and did not express concerns about the quality or accuracy of the information; (3) moderate, if the authors concluded with neither a negative nor positive tone and discussed the risks and benefits of the information or (4) varied, if it was explicitly stated that the information evaluated was of variable quality or accuracy^(10,26)^. All included studies evaluated quality or accuracy, and therefore, all studies were eligible for synthesis with the framework. One author (ED) classified all articles and 20 % were randomly selected to be classified by a second author (SM) for reliability, achieving 76 % agreement. Disagreements were resolved through discussion until consensus was reached.

### Risk of bias

The Academy of Nutrition and Dietetics Quality Criteria Checklist was used to conduct the risk of bias assessments^(33)^. This risk of bias assessment tool contains fourteen questions (four relevance and ten validity questions), and studies receive an overall rating of positive, neutral or negative, where a positive rating indicates low risk of bias and negative indicates high risk of bias^(33)^. Due to the design of included studies, a number of questions in the tool were not relevant. Therefore, most consideration was given to questions one, two and seven, as specified for descriptive studies in the tool’s manual for use^(33)^. For a study to receive an overall positive rating, questions one, two and seven must all have all received a positive response. If one or more of these questions was rated as neutral or negative, a neutral or negative overall score was awarded respectively. All risk of bias assessments were performed by one author (ED), and a random 20 % were independently reviewed by another author (RL) for reliability. Eighty-five percentage agreement was achieved, and disagreements were discussed until consensus was reached.

## Results

### Description of included studies

**Fig. 1:**
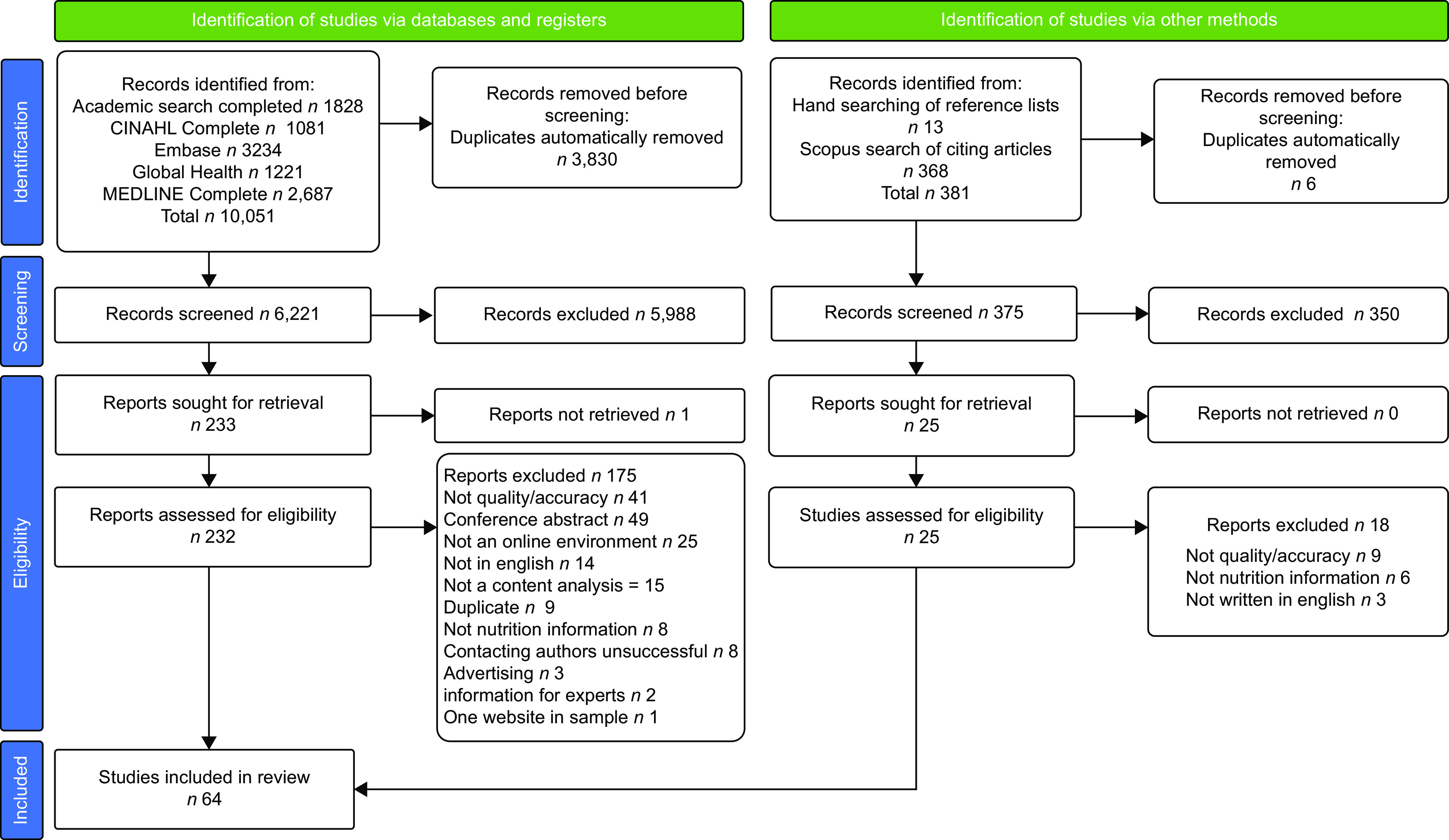
PRISMA^(29)^ flow chart detailing process of selection of literature

**Fig. 2:**
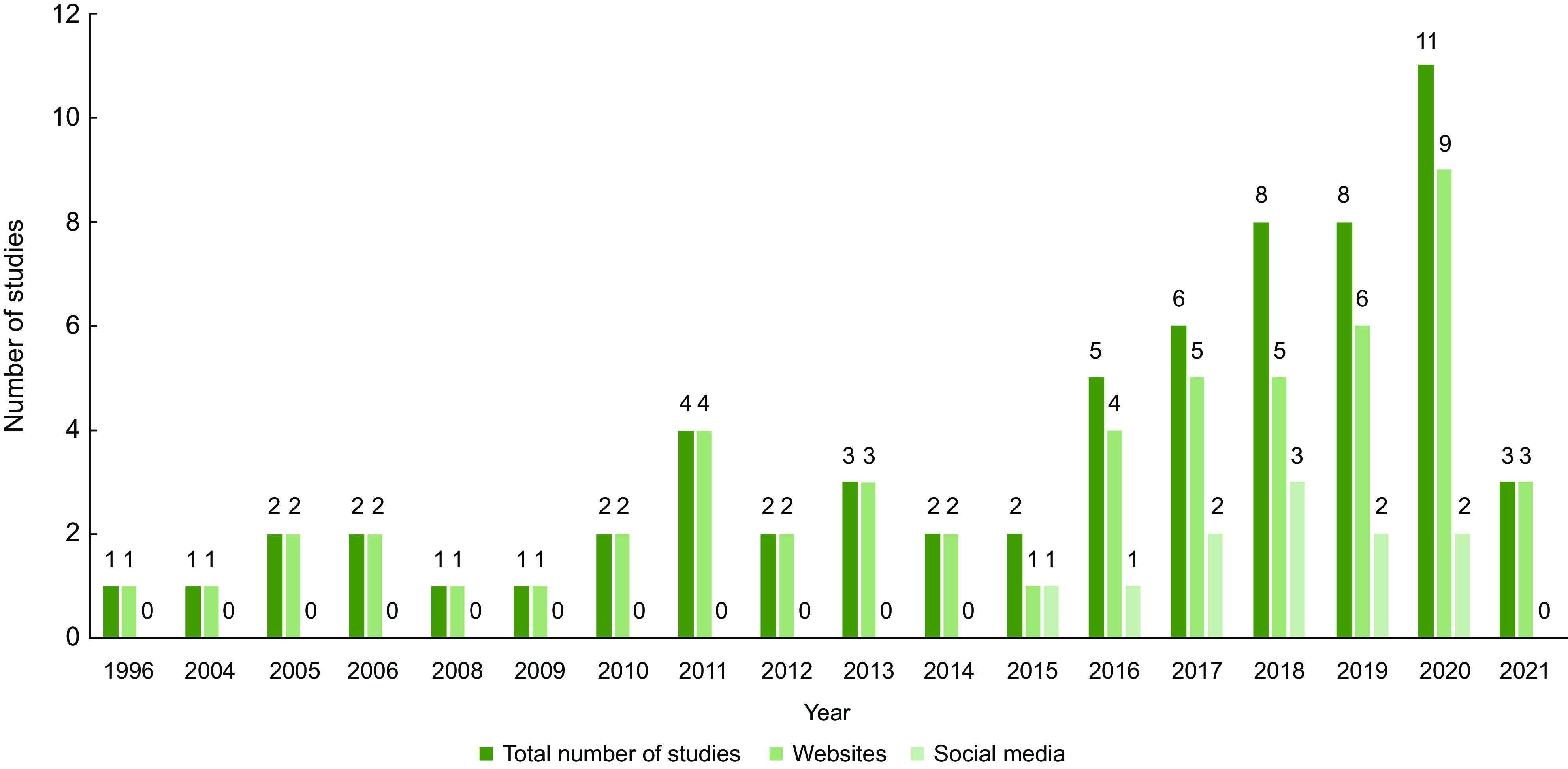
The distribution of studies examining information published on websites or social media in the final sample by year published

Sixty-four studies, published between 1996 and 2021, were included in this review (Fig. 1). The number of studies published each year shows a generally increasing trend (Fig. 2). The first study to examine social media content was published in 2015 and at least two studies per year included social media data in subsequent years, except for 2021 due to the literature searches being run in January of the same year. Reported data collection periods ranged from February 1996 to August 2020 and 16 (34·0 %) studies did not report when data were collected^(34–50)^. A summary of extracted data for studies evaluating websites and social media is provided in online Supplementary Tables 2 and 3, respectively.

Characteristics of the included studies are reported in Table 1. There was a fairly even distribution of studies that assessed quality and accuracy. The majority of included studies (82·8 %) evaluated information published on websites, and a wide range of nutrition topics were covered. Most studies (54·7 %) did not focus on information published in a specific region and those studies that did, generally evaluated information published in high-income countries. The number of websites, webpages and/or social media posts included in the studies’ samples varied greatly; the mean sample size was 165·7 (sd 359·1) and ranged from 4 to 2770.

**Table 1:** Characteristics of the content analysis studies included in the systematic review, *n* 64

Characteristics	*n*	%	References
Year published			
≥ 2020	14	21·9	^(41,44,50–52,64,65,73,88,89,112)^
2010–2019	42	65·6	^(34–40,42,45,47,53–60,66–72,74–77,81,83–87,90–93,113,114)^
2000–2009	7	10·9	^(43,46,48,61,62,78,82)^
≤ 1999	1	1·6	^(94)^
Online environment*			
Websites	53	82·8	^(34,36–41,43,45–48,50–53,55,56,58,61,62,64–69,71,73–83,86–93,112–115)^
Wikipedia	2	3·1	^(71,92)^
Social media	12	18·8	^(35,42,44,54,57,58,63,70,72,84,85,116)^
YouTube	6	9·4	^(35,42,58,63,70,85)^
Blogs	3	4·7	^(44,72,84)^
Facebook	1	1·6	^(76)^
Twitter	1	1·6	^(97)^
WhatsApp	1	1·6	^(60)^
Evaluation of			
Accuracy	23	35·9	^(36,40,42,43,51,53–55,57,60,67,68,71,74–80,115,116)^
Quality	17	26·6	^(34,35,38,39,44,47,83,86–91,93,112–114)^
Accuracy and quality	24	37·5	^(37,41,45,46,48,50,52,56,58,59,61–64,66,70,72,73,81,82,84,85,92)^
Country/countries of interest			
High income	28	43·8	^(40–42,44,48,50,51,53,54,59,61,62,65,66,72,73,76,78,80,82,84,88,89,91,93,112,113,115)^
Middle income	1	1·6	^(37)^
Low income	0	0	–
N/A – no location specified	35	54·7	^(34–36,38,39,43,45–47,52,55–58,60,63,64,67–71,74,75,77,79,81,83,85–87,90,92,114,116)^
Nutrition-related topic studied*			
Disease management	17	26·6	^(34,36,40–43,47,55,58–60,62,75,81,91,93,115)^
General	15	23·4	^(37,39,48,50,56,65,71,76,78,79,82,84,92,112,116)^
Maternal and child	10	15·6	^(38,46,51,53,61,73,74,77,80,88)^
Supplements	6	9·4	^(35,54,64,65,89,113)^
Weight loss	6	9·4	^(44,57,63,66,69,83)^
Dietary patterns	4	6·3	^(38,72,87,90)^
Disease prevention	3	4·7	^(45,81,86)^
Sports nutrition	2	3·1	^(38,67)^
Immune function	2	3·1	^(65,114)^
Food safety	2	3·1	^(52,85)^
Other	2	3·1	^(68,70)^

* Studies may fall under more than one category.

### Risk of bias assessments

Most studies were rated for risk of bias as negative (28·1 %) or neutral (51·6 %); thirteen studies (20·3 %) received an overall positive rating (online Supplementary Tables 2 and 3). Negative or neutral ratings were typically given due to risk of bias in the sample selection. For example, it was uncommon for the screening of content to involve more than one researcher and reporting of inclusion/exclusion criteria and search methods often lacked detail. Additionally, negative or neutral ratings were also given due to risk of bias in the evaluation of information quality and accuracy. For example, in three studies one rater independently performed all quality or accuracy evaluations and there was no method of measuring reliability, and thirteen studies did not report the number of raters involved.

### Quality and accuracy assessment methods

Methods used to evaluate information quality varied across the forty-one studies that assessed quality (Table 2). The most common quality assessment methods were use of study-specific criteria developed by the study authors (23·4 %), the DISCERN Instrument (17·2 %) and the JAMA Benchmarks (10·9 %). The application of the JAMA Benchmarks was consistent across the studies that used this tool; however, the application of the DISCERN Instrument varied.

**Table 2:** Methods of quality and accuracy assessment used in included studies, *n* 64

Assessment tools and methods	*n*	%
Quality* (*n* 41)		
Criteria/quality metrics developed by authors	15	36·6
DISCERN Instrument	11	26·8
DISCERN not adapted, all 16 questions, possible score of 80	2	4·9
DISCERN adapted, questions 1–15, possible score of 75	2	4·9
DISCERN adapted, average score of all questions, possible score of 5	5	12·2
DISCERN adapted, questions removed	4	9·8
DISCERN adapted, questions amended	1	2·4
JAMA Benchmarks	7	17·1
HONCode Principles	3	7·3
Criteria developed for previous study	2	4·9
Global Quality Score	2	4·9
EQIP	1	2·4
Usefulness score	1	2·4
LIDA Instrument	1	2·4
HITI Criteria	1	2·4
International Patient Decision Aid Standards tool	1	2·4
MARS	1	2·4
QWEB tool	1	2·4
Accuracy* (*n* 47)		
Assessed against authoritative guidelines	16	34·0
Assessed against academic literature	13	27·7
Assessed against national dietary guidelines	12	25·5
Professional knowledge/opinion	5	10·6
Accuracy reference not reported	3	6·4
Assessed against LID Dictionary of Metabolism and Nutrition	1	2·1
No. of raters performing quality/accuracy evaluations and reliability measures		
2, independent evaluation of entire subsample	20	31·3
Not reported	13	20·3
≥ 3, independent evaluation of entire subsample	11	17·2
1, subsample independently evaluated by second rater	5	7·8
1, reliability of use of assessment tool established before analysis	3	4·7
1, no reliability measures	3	4·7
2, simultaneous assessments	2	3·1
1, all evaluations checked by another author	1	1·6

HONCode, Health on the Net Code; JAMA, Journal of American Medical Association; Health Information Technology Institute; EQIP, Ensuring Quality Information for Patients; IPDAS, International Patient Decision Aid Standards; LIDA, MinervaLIDAtion; MARS, Mobile App Rating Scale.

* Studies may fall under more than one category.

The majority of studies evaluating information accuracy assessed correctness against authoritative guidelines (*n* 16, 34·0 %)^(41,45,46,51–63)^, academic literature (*n* 13, 27·7 %)^(36,40,43,58,64–72)^ or national dietary guidelines (*n* 12, 25·5 %)^(48,50,56,66,73–80)^. A scoring system was used for accuracy evaluations in 16 (34·0 %) studies^(37,41,46,48,55,59,60,62,63,66,70,71,75,81,82)^. Fourteen (29·8 %) studies included an evaluation of the comprehensiveness of information in accuracy assessments^(40,45,46,51,53,59–61,67,71,74,75,80,81)^. Accuracy was evaluated as a component of quality in seven (14·9 %) studies^(37,41,48,82–85)^. Forty-seven studies (67·2 %) did not mention ethics or that approval from an ethics committee was not required.

### Quality and accuracy results

Quality and accuracy coding classifications are presented in Table 3. Overall, 48·8 % of studies that investigated information quality were coded as poor. Of the studies that evaluated information quality on websites and social media, 47·1 % and 62·5 % were classified as poor, respectively. Similar proportions of studies were classified as poor, good and moderate between studies evaluating information quality on websites and social media. One study investigated websites and YouTube content and found a slightly larger proportion of low quality information on YouTube^(58)^. Higher proportions of poor classifications for quality were observed for studies evaluating information about weight loss (*n* 5, 100 %) and supplements (*n* 3, 75 %), and a greater proportion of good classifications for information about child and maternal nutrition (*n* 2, 40 %), although the number of studies that evaluated these topics was small.

**Table 3:** Quality and accuracy coding classifications of included studies, *n* 64

Coding classification	*n*	%	References
Quality, all (*n* 41)			
Poor	20	48·8	^(34,35,37,39,41,44,45,47,48,52,56,58,63,66,69,70,83,87,91,113)^
Moderate	10	24·4	^(38,50,61,62,64,72,82,85,86,112,114)^
Varied	5	12·2	^(46,59,81,89,90)^
Good	5	12·2	^(73,84,88,92)^
Quality, websites (*n* 34)			
Poor	16	47·1	^(34,37,39,41,45,47,48,52,56,58,64,66,69,83,87,91,113)^
Moderate	9	26·5	^(38,61,62,82,86,112,114)^
Varied	5	14·7	^(46,59,81,89,90)^
Good	4	11·8	^(73,88,92,93)^
Quality, social media (*n* 8)			
Poor	5	62·5	^(35,44,58,63,70)^
Moderate	2	25	^(72,85)^
Varied	–		–
Good	1	12·5	^(80)^
Accuracy, all (*n* 47)			
Poor	23	48·9	^(37,41,45,48,51,52,54,56–58,60,63–70,74,76,77,79,80)^
Moderate	12	25·5	^(36,40,53,61,62,71,72,75,82,84)^
Varied	5	10·6	^(55,59,78,81,116)^
Good	8	17	^(42,46,50,73,85,92,115)^
Accuracy, websites (*n* 38)			
Poor	18	47·4	^(37,41,45,48,51,52,56,64–69,74,76,77,79,80)^
Moderate	10	26·3	^(36,40,43,53,60–62,71,75,82)^
Varied	4	10·5	^(55,59,78,81)^
Good	5	13·2	^(46,50,73,92,115)^
Accuracy, social media (*n* 10)			
Poor	5	50	^(54,57,58,63,70)^
Moderate	2	20	^(72,84)^
Varied	1	10	^(97)^
Good	2	20	^(42,85)^

Overall, 48·9 % of studies assessing accuracy were coded as poor. Similar results were observed between studies that evaluated accuracy on websites and social media, with 47·7 % and 50 % classified as poor, respectively. One study compared the accuracy of website content with YouTube content, finding that accuracy was significantly higher for websites than YouTube (*P* < 0·0001)^(58)^. Higher proportions of poor classifications for accuracy were observed for studies evaluating information about weight loss (*n* 4, 100 %) and supplements (*n* 3, 100 %), although the number of studies that evaluated these topics was small. For some topics, there was only study available, and they had poor ratings (immune function and sports nutrition).

Findings about the quality and accuracy of information from different publishers varied between studies. Three found that government websites had lower quality scores compared with other categories, such as news sites and non-government organisations^(81,86,87)^ and one study found government sites provided the least accurate information^(81)^. Conversely, government websites received some of the highest scores for quality in four studies^(48,73,88,89)^ and accuracy in one^(51)^. Commercial websites’ information quality or accuracy was poorer than other publishers in six^(46,64,69,86,87,89)^ and four studies, respectively, while two studies found commercial entities published the highest quality information^(59,88)^, and one found commercial health channels published the most accurate information^(78)^. Blogs provided the poorest quality information in three studies^(66,88,90)^ and least accurate information in two^(53,66)^, although blogs were found to provide the most accurate information in one study^(69)^. Organisations and/or academic institutions received the most favourable quality assessments in four studies^(46,48,81,91)^ and provided the most accurate information in five studies^(46,51,53,79,81)^. Two studies evaluated information published by nutritionists and dietitians, both stating that information from dietitians was of higher quality and accuracy^(72,82)^. Two studies focused solely on Wikipedia, one was coded as good for quality and accuracy^(92)^ and one coded as moderate for accuracy^(71)^. No differences in the quality or accuracy of information by different publisher categories were observed in two and five studies^(37,93)^, respectively^(40,59,63,67,75)^.

A breakdown of results for each quality criteria was not always reported. From studies that reported results for each criteria, the most consistently reported contributor to poor quality scores was a lack of reference to the original source of information, which was reported in eleven (26·8 %) studies^(35,44,48,52,56,66,69,72,85,89,90)^. Two articles examined the correlation between information quality and accuracy, one observed a weak correlation (*r* = 0·250, *P* < 0·05)^(45)^ and one observed no correlation (*r* = 0·18, *P* > 0·05)^(62)^. In another study, almost half of the websites deemed low quality contained accurate information^(58)^.

## Discussion

This systematic review included content analysis studies that investigate the quality and/or accuracy of nutrition-related information published on websites and social media. Half of the included studies found that the quality and/or accuracy of nutrition-related information examined was suboptimal. There was some variation in quality and accuracy between nutrition-related topics but very little consistency in findings about the level of quality or accuracy from different publishers of information. These results about the online nutrition-related information are discussed and summarised into four substantive observations.

### Overall quality and accuracy

A major finding of this review was the high prevalence of poor-quality information in online environments. This finding is consistent with the outcomes from three systematic reviews that investigated the quality of health information on websites and found that online health information was of suboptimal quality^(10,26,28)^. Further, a systematic review investigating the use of social media for communicating health information found that one of the biggest limitations of using social media for this purpose was the lack of quality and reliability of health information^(94)^. A slightly higher rate of social media studies received an overall poor classification for quality findings compared with websites, which suggests that information quality may be more of a problem on social media. Further research that evaluates and compares the quality of information from both websites and social media is required to confirm if information quality is worse on social media.

Findings from the included studies indicate that that there is a large amount of inaccurate nutrition information present on websites and social media. These results are not surprising, given the widespread concerns about the prevalence and propagation of online health and nutrition misinformation^(5,6,14,15)^. Findings about accuracy in this review are also consistent with Eyesenbach *et al*.^(26)^ and Zhang *et al*.^(10)^ who included accuracy as a component of quality in their systematic reviews about health information on websites, both concluding that, overall, the standard of information was poor. Further, a systematic review investigating the prevalence of health misinformation on social media identified that diet misinformation is present in greater amounts compared with other health topics^(27)^.

### Quality and accuracy by topic

Studies that evaluated information about weight loss or supplements received a larger proportion of poor classifications about quality and accuracy findings compared with other topics. Weight loss and supplements are large commercial industries^(95,96)^. Assessment of financial and conflict of interest disclosures are a prominent component of quality assessment tools^(10)^, which may explain why these are rated more frequently as poor-quality information about weight loss and supplements. Further, the high rate of inaccuracies about these topics in online sources may mirror the high rate of misleading claims among information about products and services^(97)^. Consistent with findings about the accuracy of weight loss information in this review, Suarez-Lledo *et al*.^(27)^ found that misinformation about weight loss diets and promotion of eating disorders was present on social media in moderate amounts. Further, restrictive eating practices have been claimed as being healthy on websites and blogs^(72,76)^. Inaccurate online information about weight loss diets may be a particular concern, because diets have been identified as a risk factor for the development of eating disorders and engagement with health-related online content can contribute to poor body image, body dissatisfaction and restrictive eating^(98–101)^. Therefore, inaccurate weight loss information in online environments may exacerbate the potential for harm and warrants further investigation.

### Quality and accuracy by publisher typology

Included studies had contradictory findings about the quality and accuracy of information published by government agencies, academic institutions, blogs and commercial entities. These findings are concerning because consumers consider publishers as an indicator of nutrition information’s credibility^(102)^, and typically view organisations, academic institutions and government agencies as trustworthy, and commercial entities, Wikipedia and social media as less trustworthy when selecting health information^(103)^. As such, when selecting information consumers may perceive nutrition information as credible, even if it is poor quality or inaccurate because the publisher is considered to be credible. Findings from this review suggest that the publisher of information may not always be a reliable indicator of the quality or accuracy of online nutrition-related information and using the publisher of online information only to determine credibility may put consumers at risk of being misinformed.

### Evaluation methods and limitations of included studies

Quality and accuracy assessment methods varied between studies, particularly for studies investigating information quality. The use of a range of different quality assessment methods has also been observed in other systematic reviews and creates difficulty in comparing findings about information quality because quality principles are not consistently measured^(10,26,28)^. Some studies measured accuracy as a component of information quality, while others did not consider accuracy at all. There was little evidence of a relationship between information quality and accuracy. This suggests that quality and accuracy should both be assessed when evaluating information so that all factors are considered when drawing conclusions about the overall credibility of information.

It was common for accuracy measures to include an assessment of comprehensiveness. Some studies classified missing information in the same way as information that was inaccurate. While it is important to provide complete information^(14)^, the absence of information may not be the same as the presence of inaccurate information. Accuracy measures that did not distinguish between inaccurate and incomplete information may have overstated the presence of inaccurate information. Differing considerations about information completeness in accuracy measures of included studies may account for some of the variation in conclusions about publishers of accurate and inaccurate information in this review. In future studies, use of accuracy measures that evaluate comprehensiveness should clearly distinguish between missing and inaccurate information.

Many of the studies included in this review had common limitations. First, most studies did not address ethical issues in their design or reporting. While ethics approvals may not have been required due to the use of publicly available data, research in online environments including social media can involve ethical issues such as identifying included websites or social media profiles, particularly if those sites or profiles identify individuals. Second, it was also rare for more than one researcher to be involved in the sample screening, which is a potential source of bias. Future studies about online health or nutrition information should aim to minimise the risk of bias by involving more than one researcher in screening. Finally, no included studies that evaluated the quality of social media content used tools that were developed specifically for social media. Use of quality assessment tools that have not been designed for social media may be inappropriate to measure information from social media, due to the many unique characteristics of social media platforms that may not be considered, such as the use of brief information and covert advertising^(104,105)^. A quality assessment tool for social media-based health information has recently been developed that considers social media’s characteristics^(105)^ and recommended for future studies examining the quality of health information on social media.

### Strengths, limitations and directions for future research

This systematic review has several strengths, including the large number of studies included (*n* 64) and wide range of nutrition-related topics examined. Further, it provides an analysis of research examining the quality and accuracy of online nutrition-related information since the Internet became widely available. This review also has limitations. First, a number of studies that examined information related to a broad health topic that encompassed nutrition were excluded because data specific to nutrition could not be extracted. In these instances, authors were contacted; however, most did not provide data. Second, readability is often considered a component of quality^(10)^; however, no search terms related to readability were included in the search strategy. Such terms were not included because it is common for studies to focus only on readability, and studies that only considered one aspect of information quality were beyond the scope of this review. Third, although the risk of bias assessment tool used was the most appropriate option available, there were several items that were not relevant, and the application of the tool was modified for the purposes of this review. Finally, due to the different assessment methods employed in the included studies, a previously established coding framework was used to assist in the interpretation of findings. Findings were coded based on the authors’ overall tone about the quality or accuracy of information; however, the interpretation of results was not always consistent between studies and in some studies the authors’ tone about accuracy was poor due to information being incomplete, rather than inaccurate. Additionally, agreement in coding decisions was low (76 %); however, coding disagreements were mainly between varied and moderate categorisations and therefore were not likely to significantly impact the findings.

Findings from this review have implications for future research and practice. Few studies investigated the quality or accuracy of nutrition information on social media, and some popular social media platforms, such as TikTok and Instagram, are yet to be studied. Future research should focus on social media, particularly platforms that have not been evaluated. Online health misinformation is a complicated problem and effectively combatting it will likely require a range of solutions. Improving the eHealth and media literacy of consumers may be one such solution; however, more research about how eHealth and media literacy skills can be improved in various demographic groups is needed to inform future policy actions^(106)^. Greater regulation and moderation of information published on online platforms have also been identified as a possible solution, particularly on social media^(107)^. There has been a push for social media giants to accept greater responsibility for the publication and propagation of harmful health misinformation on their platforms; however, thus far, there has been limited progress^(107,108)^. Communication has been identified as a core nutrition competency and harnessing the Internet and social media for efficient, effective nutrition communication is recommended in Australia’s decadal plan for nutrition^(109,110)^. Nutrition professionals and experts can counteract nutrition misinformation by publishing accurate and high-quality nutrition information online and avoiding common mistakes, such as the omission of reference to original source material. Utilising methods such as search engine optimisation, to ensure that credible information is visible, and referring to resources such as *Guidance for Professional Use of Social Media in Nutrition and Dietetics Practice* ^(111)^, to ensure information is of high quality, are also recommended.

## Conclusion

This systematic review found that poor-quality and inaccurate nutrition-related information is prevalent on websites and social media. These high rates of suboptimal nutrition-related information are concerning because the public is increasingly relying on the Internet to source information about food and nutrition and are likely to encounter misleading information when using the Internet for this purpose. Results from this review also indicate that the publisher of information is not a good indicator of its quality or accuracy. Consumers typically use information’s publisher as an indicator of its credibility, which puts them at a greater risk of being misinformed when seeking nutrition information online. Future research should investigate methods to improve the public’s eHealth and media literacy to lessen the potential for harm caused by nutrition- and health-related misinformation. To improve the quality and accuracy of nutrition-related information available on websites and social media, credentialed experts and nutrition professionals should publish and promote their own high-quality and accurate information, and greater moderation, regulation and fact-checking of information should be carried out by social media companies and other online platforms that publish nutrition- and health-related information.

## Supplementary material

For supplementary material accompanying this paper visit https://doi.org/10.1017/S1368980023000873. click here to view supplementary material
