# Supplementary material for: Quality and accuracy of online nutrition-related information: a systematic review of content analysis studies
Source: Public Health Nutr. 2023 May 4;26(7):1345–57. doi: 10.1017/S1368980023000873 (PMC10346027; doi:10.1017/S1368980023000873)
Supplement: Supplementary file 1 [file S1368980023000873sup.zip › S1368980023000873sup001.docx]

Supplementary Table 2: Study characteristics, results and risk of bias assessments of content analysis studies evaluating quality and/or accuracy of nutrition-related information on websites

| **Study, year** | **Nutrition-related topic** | **Country** | **Sample size** | **Data collection period** | **Search & selection strategy** | **Evaluation method/tool(s) used** | **No. of raters** | **Inter-rater reliability assessed?** | **Key findings** | **Risk of bias assessment** |
| --- | --- | --- | --- | --- | --- | --- | --- | --- | --- | --- |
| **Quality** | | | | | | | | | | |
| Ng et al.^(54)^ 2021 | Dietary supplements for weight loss | Australia, Canada, UK, US | 56* | August 2020 | Google search (Australia, Canada, US and UK sites each used)  Search terms: “dietary supplements for weight loss”, “herbal medicine for weight loss”, “herbs for weight loss”, “natural health products for weight loss”, “natural products for weight loss”, “supplements for weight loss”  First 20 results for each search screened | DISCERN Instrument | 2 | No | - Mean question 16 score 2.7 ± 1.1 (max. possible score of 75)  - Minimum question 16 score 1.5, max. 5  - Mean overall score 45.1 ± 13  - Minimum overall score 28, maximum 67.5  - Government websites had the highest mean DISCERN score of 64.3 and commercial had the lowest mean scores at 38.0. | Positive |
| Lobo et al.^(52)^ 2020 | Pregnancy | Australia | 136 | August 2018 | Google search  Search terms:  "nutrition and pregnancy"  "food safety during pregnancy" "supplements during pregnancy" "weight gain during pregnancy" "symptoms during pregnancy" | DISCERN Instrument | 2 | No | - 81 (59.6%) websites scored 5 on DISCERN (max. possible score of 5)  - 29 (23.3%) websites scored 4  - 25 (18%) websites scored 2 or 3  - 1 (0.7%) website scored 1  - 100% of business/company sites, 95.5% of government sites, 93.8% of organisational sites, 90% of factsheets, 85.7% of research articles, 76.5% of organisational, 50% of other or unclear sources, 40% of community groups and 0% of blogs scored 4 or 5 on DISCERN | Neutral |
| Ruan et al.^(56)^ 2020 | General nutrition (for adolescents) | Australia | 22 | August 2018 | Google search  Search terms: 12 search terms for adolescent’s health information used  First five pages of results screened | Adapted DISCERN Instrument | 2 | Yes  NR | - Average DISCERN score: 49.6 ± 13.6 (max. possible score of 75) | Positive |
| Alfaro-Cruz et al.^(34)^ 2019 | Irritable bowel syndrome | None | 74 | NR | DuckDuckGo and Ixquick searches  Search terms: "IBS, diet, children" and "IBS, diet, adults"  First 30 websites identified and screened. | DISCERN Instrument | 3 | Yes  Intraclass Correlation Coefficient (95% CI)  Children: 0.93 (0.86 - 0.97)  Adults: 0.79 (0.61 - 0.89) | - Average overall DISCERN score for information for children 2.1 ± 0.98 (max. possible score of 5)  - Average overall DISCERN score for information for adults 3.0 ± 0.14 | Neutral |
| Cassa Macedo et al.^(66)^ 2019 | Immune function | None | 163* | November 2018 | Google search  Search terms: "boost immunity"  First 204 results screened | JAMA Benchmarks | NR | No | - JAMA Average 2.67* ± 1.08 (max. possible score of 4)  - 4* websites had a JAMA score of 0  - 25* websites had a JAMA score of 1  - 33* websites had a JAMA score of 2  - 61* websites had a JAMA score of 3  - 40* websites had a JAMA score of 4 | Neutral |
| El Jassar et al.^(68)^ 2019 | Vegan diet | None | 67 | July 2018 | Google, Bing, Yahoo searches  Search terms: "Vegan diet" | DISCERN Instrument | 2 | No | - The overall DISCERN rating was “fair”, mean 41.6 ± 15.4 (max. possible score of 80)  - 9 (13.4%) websites rated as “excellent”, 6 (9%) “good”, 21 (31.3%) “fair”, 22 (32.8%) “poor” and 9 (13.4%) “very poor”  - Average score for blogs was 26 (“very poor”), 28.3 (“poor”) for magazine and newspaper sites, 36 (“poor”) for charitable sites, 39.7 (“fair”) for commercial sites, 47.3 (“fair”) for support sites, 60.7 (“good”) for institutional sites and 70.5 (“excellent”) for online encyclopedias  - DISCERN scores for online encyclopedias and institutional websites had no statistically significant difference between them, but they were both significantly higher than mean DISCERN scores achieved in all other website categories (P <0.05) | Positive |
| Kriz et al.^(77)^ 2019 | Weight loss  (coconut oil) | None (written in German) | 25 | February 2018 – March 2018 | Google search  Search terms: “coconut oil superfood,” coconut oil healthy,” “coconut oil lose weight”, and “coconut oil weight reduction”  First page of results screened | ***Adapted QWEB tool | NR | No | - The median overall quality score was 44.2%  - The lowest score was 11.7% and highest was 80%  - 4% (n = 1) article achieved over 75% to be considered high quality/reliable | Neutral |
| Shanahan et al.^(47)^ 2019 | Orofacial granulomatosis (dietary interventions) | None | 4 | NR | Google, Yahoo and Bing searches  Search terms: “benzoate and cinnamon free diet”, “benzoate and cinnamon exclusion diet”, “orofacial granulomatosis diet”, and “E210 E211 E212 E213 E214 E215 E216 E217 E218 E219 free diet”  First 100 results screened | DISCERN Instrument  JAMA Benchmarks | 2 | Yes  96% agreement | - Average JAMA score was *0.75 (max. possible score of 4)  - No websites were rated as “good” or “excellent” by DISCERN  - ^Minimum DISCERN score was 17, maximum was 36 (max. possible score of 80)  - ^Average DISCERN score was *26.1 "poor" | Neutral |
| Smekal et al.^(86)^ 2019 | Renal diet | Australia, Canada, UK, US | 81 | September 2018 | Google, Bing and Yahoo searches  Google searches conducted with preferences set to Australia, Canada, the UK and US  Search terms: "chronic kidney disease" | Adapted DISCERN Instrument  HONCode Principles  Adapted LIDA Instrument | 2 | Yes  (LIDA = 93.1%, κ = 0.52; HONCode = 97.7%, κ = 0.81; DISCERN = 90%, κ = 0.68) | - Average DISCERN % was 68.6 ± 12.7, minimum 35.6, maximum 87.8  - Average Reliability (LIDA) % was 67.3 ± 13.9, minimum 25.9, maximum 94.4  - Average HONCode % was 76.3 ± 17.2, minimum 32.1, maximum 100  - 9.9% websites had DISCERN, reliability and HONCode scores below 50%  - 56.8% websites had DISCERN and reliability scores between 50% and 75%  - 33.3% of websites had DISCERN and reliability scores above 75%  - 27.2% of websites had HONCode scores between 50% and 75%  - 63% of websites had HONCode scores above 75%  - There was minimal difference between DISCERN, reliability and HONCode scores between website types. However, academic/professional and non-profit sites scored slightly higher than commercial sites across all three measures* | Positive |
| Baudischova et al.^(62)^ 2018 | Supplements | Czech Republic | 850 | July 2015 – November 2015 | Seznam, Google and Centrum searches  Search terms: names of the top selling supplements in the Czech Republic  First five links from each search included | Criteria developed based on mandatory reporting legislation set by European Parliament and of the Council. Two sets of criteria were evaluated: clinical (information relevant to consumers and health professionals) and; regulatory (information required by law) | NR | No | Regulatory criteria:  - 96 (11.3%) of websites fulfilled all criteria  - 31% of websites gained more than three points (max. possible score of 6)  - Average score per page was 3.85 ± 1.35  Clinical criteria:  - 1 website met all the criteria  - The average number of points per page was 4.24 ± 1.55 (max. possible score of 10)  - Almost 44% of websites gained more than five points (exact figure not reported) | Negative |
| Aslam et al.^(61)^ 2017 | Antioxidants | None | 144 | December 2015 | Google search  Search term: "antioxidants"  First 200 results were screened | JAMA Benchmarks | NR | No | - Median JAMA score was 2 (IQR [1, 3])  - News sites had the highest JAMA scores  - Commercial and government websites had the lowest JAMA scores  - Median JAMA score for websites displaying HONCode certification was 2.5 (IQR [2, 4] but the difference was not significant (P = 0.0525) | Neutral |
| Herth et al.^(69)^ 2016 | Cancer | Germany | 60 | April 2015 | Google search  Search term: “cancer diet”  First 100 results screened | Criteria developed by authors based on previous quality assessment tools | 2 | No | - Self-help group websites average score: 21.7 (Score range 0 – 100 where a lower score indicates higher quality)  - Non-profit website average: 34.9  - Profit websites average: 59.6  - Newspaper websites average: 60.9  - Practice websites average: 63.6  - Self-help and non-profit sites consistently gave the highest quality ratings and profit orientated sites provided lower quality information  - No website fulfilled all criteria  - 13% of websites provided trustworthy and relevant information | Neutral |
| Gkouskou et al.^(38)^ 2011 | Mediterranean diet  Sports nutrition  Dysphagia in children | None | 99 | NR | Yahoo and Google searches  Search terms: “Mediterranean diet”, “sports nutrition”, “nutrition AND dysphagia AND children”  The first 20 results for each search were screened | DISCERN Instrument (first section, 8 questions only) and EQIP tool | 5 | No | *Mediterranean diet:  - EQIP average score 59%  - DISCERN average score 24.1 (max. possible score of 40)  *Sports nutrition:  - EQIP average score 55%  - DISCERN average score 23.8  *Nutrition and dysphagia in children:  - EQIP average score 42%  - DISCERN average score 24.4 | Neutral |
| Guardiola-Wanden-Berghe et al.^(39)^ 2011 | General | None | 354 | NR | Google Spain searched  Search term: "diet"  A random sample of search results and the first two pages of results were screened | Quality variables were developed by authors based on numerous previously developed quality criteria and recommendations (Health Information Locator, Dublin Core, HONCode, the Web Medica Acreditada and Netscoring) | 2 | No | - Overall mean quality score 7.05 ± 0.15 (CI 95% 6.76 -197.34) (max possible score of 22)  - Maximum score awarded was 17 and median was 7  - Websites on anorexia and bulimia were also assessed and had higher quality scores (P <0.001) than diet websites | Positive |
| Hirasawa et al.^(71)^ 2012 | Mediterranean diet | None | 32 | February 2010 | Google, Yahoo, MSN, AOL, Ask and Altavista searches  Search term: "Mediterranean diet"  First 30 results from each search engine screened | DISCERN Instrument, JAMA Benchmarks, HONCode principles | 2 | No | DISCERN:  - Mean DISCERN Score 33.8 ("poor") (max. possible score of 80)  - 22 (69%) sites categorised as "poor" or "very poor”, no sites rated as "excellent" and 1 site "good"  - On average support sites scored 39.3 ("fair") rating, institutional 36.0 ("poor"), commercial sites 27.0 ("poor")  - Institutional and commercial were significantly (P <0.05) lower than support sites.  JAMA:  - 2 (6%) sites met two benchmarks, 1 (3%) met two benchmarks, 12 (38%) met one benchmark, 17 (53%) met no benchmark  - 0 websites met all benchmarks  HONCode:  - Mean score was 3.2 (max. possible score of 8)  - 1 site (3%) met all 8 principles, 2 (6%) met 7 principles, 3 (9%) met 6 principles, 2 (6%) met 5 principles, 24 (75 %) met <5 principles | Neutral |
| **Accuracy** | | | | | | | | | |  |
| Buseck et al.^(95)^ 2021 | Coeliac disease | US | 55 | June 2019 | Google search  Search simulated for location of 20 major US cities  Search term: “coeliac disease blogs”  First 50 results from each city’s search recorded  Three additional websites included a priori | The first five claims on each website were identified and independently evaluated for accuracy by two of the authors. Accuracy assessments based on professional opinion of raters. | 2 | No | - 212 health claims relating to gluten, gluten-free diets and celiac disease were identified, from 44 websites  - 205 (97%) claims were evaluated as accurate  - 7 (3%) claims were evaluated as inaccurate or lacking sufficient evidence  - Inaccurate or unproven claims were present on personal, recipe or commercial sites and no inaccurate or unproven claims were present on national organisation sites | Negative |
| Cannon et al.^(96)^ 2020 | Pregnancy | Australia | 18 | November 2017 | Google, Yahoo and Bing searches  Search terms: "Nutrition AND Pregnancy"  First 6 pages of results were screened, and information published by government organisations was included. | **Information was compared to the Australian Government’s Healthy Eating During Pregnancy Guidelines (16 evidence-based guidelines) | 2 | No | - On average websites provided accurate information on 8 out of the 16 recommendations (50%).  - The most accurate and informative website provided information on almost all (15/16) recommendations.  - The least accurate and informative websites provided information on few (3/16) of the recommendations. | Neutral |
| Rachul et al.^(55)^ 2020 | Supplements (Immune function, COVID-19) | Canada & US | 227 | April 2020 | Google search (repeated for Canada and US location settings)  Search terms: “boost immunity” AND “coronavirus” | The literature states that vaccines are the only way to increase immunity against COVID-19.  Any claim that consumption or avoidance of a supplement to boost immunity against COVID-19 was considered inaccurate. | 2-3 | Yes  Cohen’s Kappa = 0.90 | - 11.9% of websites noted that there is a lack of evidence to support the role of supplements in boosting immunity (correct)  - 40% of websites stated that supplements could boost immunity (incorrect)  - 34.8% of websites stated that Vitamin C could boost immunity (incorrect)  - 34.4% of websites stated that diet could boost immunity (incorrect)  - 27.2% of news sites and 58.9% of commercial sites stated that supplements could boost immunity (incorrect) | Neutral |
| Sidnell & Nestel ^(57)^ 2020 | Pregnancy | UK | 130 | August 2018 – December 2018 | Google search  Internet modelling report used to identify top 12 search terms used for pregnancy-related nutrition information  First two pages of results for each term screened | **Information compared to the NHS advice about: foods to avoid, foods to eat and supplement use during pregnancy  Information was classified as accurate, inaccurate or missing | 1 | No | - 83 (64 %) websites contained a mix of accurate and inaccurate advice  - 23 (18 %) websites were complete and accurate  - 21 (16 %) websites were entirely inaccurate  - 3 (2%) websites lacked any relevant advice  - When websites were grouped as commercial and not-for-profit, commercial websites were significantly less accurate than not-for-profit websites (median percentage difference –8·0, 95 % CI –29, 0·0, P< 0·02) | Neutral |
| Bernard et al.^(63)^ 2018 | Type 2 Diabetes | None | 42 | May 2016 | Google, Yahoo and Bing searches  Search terms: "diabetes diet", "how to eat with diabetes", and "medical nutrition therapy for diabetes"  Top 25 results for each term screened | A scoring tool based on guidelines from the American Diabetes Association, American Academy of Diabetes Educators, and the Academy of Nutrition and Dietetics was developed for this study | 3 | Yes  Intraclass correlation coefficient  r = 0.994 (95% CI 0.986- 0.997) | - Mean accuracy score for all websites was 11.4 points ±7.75 (max. possible score of 30)  - Scores ranged from 2 to 29 points | Neutral |
| Htet et al.^(73)^ 2018 | Polycystic Ovary Syndrome (dietary management) | None | 15 | August 2016 | Google, Bing, Yahoo searches from Australia, UK and US domains  Search terms: "pcos", "polycystic ovary syndrome", "polycystic ovarian syndrome"  First two pages of each search results screened | ** 11 item accuracy checklist developed based on the ADG and US Dietary Guidelines for Americans. One point given for missing information, 2 points for partially complete, 3 points for comprehensive and accurate information, -1 for inaccurate information. | 2 | No | - Mean diet accuracy scores of 23 ± 6 (possible scoring range of -11 – 33)  - Diet accuracy scores ranged from 13 - 33  - 60% of websites linked to or referenced dietary guidelines for PCOS  - No inaccurate information present  - No significant association between diet accuracy score and website type | Positive |
| Khanna et al.^(75)^ 2018 | Dermatology | None | 30 | December 2017 | Google searches  Search terms: “diet and acne”, “diet and psoriasis”, “diet and eczema”  First 10 results from each search screened | Information was compared to current literature available on PubMed and MEDLINE | NR | No | Acne:  - 9 (90%) websites recommended avoiding dairy (accurate)  - 9 (90%) recommended avoiding high GI foods (accurate)  - 7 (70%) websites recommended omega-3 fatty acids and antioxidants (no evidence)  Psoriasis:  - 7 (70%) recommended avoiding alcohol (no evidence)  - 6 (60%) recommended avoiding gluten (accurate if individual has coeliac specific antibodies)  Eczema:  - 8 (80%) recommended avoiding dairy (no evidence)  - 5 (50%) recommended avoiding soy and wheat/gluten (no evidence) | Negative |
| Ramachandran et al.^(83)^ 2018 | General | Australia | 9 | September 2017 | The most "liked" Australian Facebook Pages were identified through marketing company Socialbakers. The website listed for each page was included | Information compared to the AGHE | 2 | No | - 2 (22.2%) websites aligned with 9 AGHE categories (AGHE contains 9 categories)  - 3 (33.3%) websites aligned with 8  - 1 (11.1%) aligned with 5  - 1 (11.1%) aligned with 4  - 1 (11.1%) aligned with 3  - 1 (11.1%) aligned with 2 | Neutral |
| Jimenez-Liñan et al.^(40)^ 2017 | Gout | Australia, Canada, UK, US | 85 | NR | Google searches for Australia, Canada, UK and US  Search terms: "gout" (first 200 pages for US, UK, first 50 for Aus and Canada), “gouty arthritis,” “gout diagnosis,” and “gout treatment” (first 50 results from all Google domains) | **A checklist of 19 key information points about gout and nutrition was developed by the authors. Information was assessed against the checklist for accuracy and comprehensiveness. | 2 | Yes  79.5% agreement | - Diet and uric acid load: 15% of websites provided accurate information, 6% inaccurate and 79% no information  - Obesity as a risk factor for hyperuricemia: 67% provided accurate information, 18% inaccurate, 15% no information  - Dietary and lifestyle factors as a cure for gout: 80% provided accurate information, 11% inaccurate, 9% no information. | Neutral |
| Storr et al.^(87)^ 2017 | Pregnancy | None | 693 | July 2014 | Google Australia, Google Blogs, Yahoo Australia, Bing searches  Search terms: 64 search terms identified through a Google Trends study on information seeking during pregnancy  Results from the first 2 pages were included | **Assessed against the 2013 ADG and RDI of energy, protein, folate, iodine and iron. | NR | No | - 275 (39.7%) considered accurate  - 260 (37.5%) considered inaccurate  - 158 (22.8%) contained a mix of both accurate and inaccurate information  - Government websites had the highest percentage of accurate (46.1%) and mixed (31.5%) information  - New Zealand websites had the highest percentage of accurate information (66.7%).  - Indian websites accounted for the highest percentage of mixed information (38.5%)  - American websites had the highest percentage of inaccurate information (46.7%) | Neutral |
| da Silva Gomes Monteiro et al.^(67)^ 2016 | General (children under 2) | None (written in Portuguese) | 50 | August 2014 | Google searches  Search terms: "infant feeding", "complementary feeding", "baby feeding", and "first baby food" | **Evaluated against the Food Guide for Children Under Two Years of the Brazilian Ministry of Health | 1 | No | - All websites included were classified as unscientific and included blogs, food companies' websites and websites specialising in child nutrition  - 5 (10%) correctly presented all 10 steps included in the Guidelines  - Exclusive breastfeeding for 6 months: 80% accurate, 18% inaccurate and 2% not mentioned  - Complementary feeding from 6 months: (36) accurate, 12 (24) inaccurate, 20(40) not mentioned  - Food offer: 64% accurate, 26% inaccurate, 10% not mentioned  - Meal times according to that of the family: 30% accurate, 10% inaccurate, 60% not mentioned  - Consistency: 48% accurate, 26% inaccurate, 26% not mentioned  - Varied food: 66% accurate, 4% inaccurate, 30% not mentioned  - Daily offer of fruit and vegetables: 60% accurate, 4% inaccurate, 36% not mentioned  - Avoid offering processed foods: 36% accurate, 4% inaccurate, 60% not mentioned  - Hygiene: 26% accurate, 0 inaccurate, 74% not mentioned  - Encourage sick children to eat: 12% accurate, 0 inaccurate, 88% not mentioned | Neutral |
| Hoffman et al.^(72)^ 2016 | Hydration & exercise | None | 141 | December 2014 | Google search  Search terms: "hydration and exercise", "hydration guidelines and exercise", "drinking fluids and exercise", "drinking guidelines and exercise"    First 50 websites for each search were screened | **Assessed against 6 statements from current literature about hydration during exercise | 2 | No | - 69.5% of websites accurately stated that some weight loss should occur during exercise  - 7.3% accurately indicated that fluid consumption should be based on thirst  - 10.4% accurately mentioned that electrolyte intake is not generally necessary during exercise.  - It was mentioned that dehydration is generally not a cause of heat illness (3.4% of websites) or muscle cramps (2.4% of websites)  - 43.3% accurately reported that overhydration is a risk factor for exercise-associated hyponatremia | Neutral |
| Temple & Fraser ^(88)^ 2014 | General (common nutrition misconceptions) | None | 22* | November 2013 | Google searches for Wikipedia entries that covered information related to the 32 statements of "common misconceptions" related to nutrition. | Information was compared to an accuracy statement that was developed based on the literature and coded based on level of accuracy | NR | No | - 7 (54%) topics scored 5 for accuracy (max. possible score of 5)  - 2 (15%) topics scored 4.5 for accuracy  - 3 (23%) topics scored 4 for accuracy  - 1 (8%) topic scored 3.5 for accuracy  - Average accuracy score for nutrition-related topics was 4.58 | Negative |
| Agricola et al.^(59)^ 2013 | Pregnancy | Italy | 226 | October 2011 | Google search  24 search terms were developed based on suggestions from women planning pregnancy and relevant health professionals  First 10 results, excluding Google ads were screened | **A checklist of 19 items based on guidelines from the American Journal of Obstetrics and Gynaecology. Information was assessed to determine if it was: a) absent, incorrect or incomplete; or b) correct and complete. | 1 | No | - 89 websites (39.4%) provided accurate and complete information about folic acid supplementation before pregnancy  - 60.6% of websites' information about preconception folic acid supplementation was absent, incorrect or incomplete.  - Most of the correct recommendations were significantly less represented in websites belonging to the 'Communities and blog' category compared with those belonging to the 'Medical/Public Agency' category: folic acid supplementation (OR 0.254; CI 0.098-0.664; p = <0.01) | Negative |
| Dawson et al.^(36)^ 2011 | Cancer (breast cancer and lymphedema) | None | NR | NR | Google search  Search terms: "lymphoedema", "lymphedema", "lymphatic dysfunction", "diet", "exercise", "cure", "management", "breast cancer". | Compared to peer-reviewed literature | NR | No | - *5 websites provided incorrect or misleading information  - *6 websites provided accurate information | Negative |
| Post et al.^(82)^ 2010 | Type 2 Diabetes | None | 39 | March 2010 | Google and Yahoo searches  Search terms: “diabetes and nutrition”  First 100 results from each search were screened  Internal links to other pages within the same included website were also screened | **Information was compared to ADA recommendations for diet to determine if it explicitly matched the ADA guidelines. | NR | No | - Mean matching score for websites was 3.56 ± 2.20 (max. possible score of 11)  - Many of the websites did not provide inaccurate information, but rather, had important pieces of information that were not included | Negative |
| Michael et al.^(43)^ 2009 | Kidney stone prevention | None | 458 | NR | Google search  Search terms: “kidney stones and dietary recommendations”  500 websites were screened | **Information was compared to four dietary recommendations from the literature for the prevention of kidney stones | NR | No | - 80 (17.4%) of websites contained accurate information  - 10 (2.2%) contained incorrect information  - 365 (79.5%) contained deficient information (information that was not considered comprehensive)  - 3 (0.7%) contained incorrect and deficient information  - Urology and nephrology sites provided correct information on 41% and 51% of the sites, respectively. All the other types contained correct information on 13% -16% of the examined sites (P<0.001) | Negative |
| Ostry 2008 et al.^(93)^ | General | Canada | 2770 | October 2003 – November 2004 | The most used websites in Canada for nutrition and health were identified through Media Matrix web-tracking service. Sites that accounted for >90% of the time spent at health websites by Canadians were identified. A smaller subsample was selected comprising the three site categories that accounted for ~60% of the visits to the top 50 health sites. Sites were searched using French and English nutrition-related terms to find relevant pages. Pages included were randomly selected from the 8 sites identified for this study. | Information was compared to the Canada Food Guide. Information was coded as purely congruent, purely incongruent, mixed or not able to assess using the Canada Food Guide. | 2 | Yes | - 858 (31%) of pages were purely congruent with the Canada Food Guide  - 284 (10.3%) of pages were purely incongruent  - 688 (25.8%) of pages provided a mixture of congruent and incongruent information  - 9440 (33.9%) of pages provided information that could not be assessed against information from the Canada Food Guide  - A greater proportion of commercial health channel sites provided information that was congruent with the Canada Food guide than other sources | Positive |
| Davison et al.^(94)^ 1996 | General | None | 167 | February 1996 | Netscape Navigator searched  Search terms:  "Diet', "food" and "nutrition" | Information was compared to The Canadian Guidelines for Healthy Eating and Nutrition Recommendations for Canadians | 2 | Yes  99% agreement | - 76 (45.6%) of websites provided information that was consistent with the Canadian guidelines  - All academic institution websites provided information consistent with the guidelines  - 44 (57%) of private vendor sites, 31 (40.8%) of individual home pages and 1 (1.3%) of health organisation sites provided information inconsistent with the guidelines | Neutral |
| **Quality & accuracy** | | | | | | | | | |  |
| Hopkins et al.^(51)^ 2021 | Breastfeeding | Australia | 31 | May 2019 – August 2019 | Google, Bing and Yahoo searches  Search terms: "Aboriginal Breastfeeding resources", "Nutrition information for breastfeeding Indigenous Mothers", "Lactation support for Indigenous Australians", "Lactation support for Aboriginal and Torres Islanders"  First three pages of search results screened | Quality:  DISCERN Instrument (adapted for study)  **Accuracy:  Assessed against NHMRC Infant Feeding Guidelines. information was considered to have shortcomings if the first three recommendations were absent or information that contradicted them was included. | 2 | No | Quality:  - 68% (n = 21) sites scored 5 on the DISCERN Instrument and 9.8% (n = 3) scored 4 overall.  - 77.8% (n = 24) scored 4 or 5 and were considered to be of high quality.  - 19% (n = 6) scored 3, 0% scored 2 and 3.2% (n=1) scored 1.  - Governmental websites were of highest quality with 9 out of the 10 scoring a DISCERN score of 5, while online videos were of lowest quality with 3 out of the 4 scoring a DISCERN rating of 3 or less.  Accuracy:  - 74% of sites (n = 23) were considered accurate because the first three recommendations were present and accurate. | Neutral |
| Keaver et al.^(41)^ 2020 | Cancer | Ireland | 4 | NR | Websites of HSE and HSE cancer centres, cancer charities and support groups were searched  Search terms: “Cancer patient”, “Cancer survivor”, “Cancer and diet”, “Cancer and nutrition”, “Cancer and weight”, “Cancer and alcohol”, “Cancer and supplements” and “Cancer and fad diets” | ***Quality:  Adapted version of the IPDAS tool  Accuracy:  A subscore of the quality score assessed accuracy based on WCRF recommendations for cancer prevention | 4 | Yes  95% agreement | Quality:  Overall quality score average: 21.8 ± 3.0 (out of a maximum 40)  ^Accuracy:  Average accuracy subscore: 2.6 ± 0.3 (out of a maximum of 5) | Positive |
| Neunez et al.^(53)^ 2020 | Supplements (probiotics) | None | 150 | July 2018 | Google search  Search term: "Probiotics"  First 200 websites assessed for eligibility | Quality:  JAMA Benchmarks  Accuracy:  Claims made about probiotics were compared to information available in the Cochrane library. | 2 (quality only) | No | Quality:  - The median JAMA score was 3, IQR [2.5, 4] (max. possible score of 4)  - Commercial websites had the lowest JAMA score, with a significantly lower median than professional (P <0.05), health portal (P <0.005), and news websites (P < 0.0001)  Accuracy:  - 77/325 online claims (23%) were supported by substantiated scientific evidence  - 66/325 online claims (20%) were not supported by any scientific evidence | Neutral |
| Young et al.^(58)^ 2020 | Food safety | None | 625 answers from question-and-answer sites (from 160 questions) | November 2018 – May 2019 | Yahoo! Answers, Quora, Reddit, and Stack Exchange sites searched  21 search phrases used in each platform and snowballing to gather sample | Quality:  Use or references and credibility of sources used was recorded.  Accuracy:  Information was compared to information from Health Canada's Safe Food Handling Tips website and the US FoodSafety.gov website | 2 | Yes  ĸ = 0.86 | Quality:  - 10% of answers cited the source of information  - 77% of the cited sources were considered credible  Accuracy:  - 41% of answers were correct  - 39% of answers were incorrect  - 20% of answers were partially correct  - Top-rated answers had 2X higher odds of being more correct than other answers to the same question | Neutral |
| Zarnowiecki et al.^(50)^ 2020 | General nutrition (for children) | Australia, New Zealand, UK, Canada,US | 15 | NR | Google search with settings for each region: Australia, New Zealand, UK, Canada and the US.  Search terms: concept 1: lunch, lunch box or school lunch box and concept 2: ideas, tips, health(y), school, planner or planning tool  Four searches for each region conducted and first 10 pages of results screened | Quality:  MARS was adapted to assess the quality of websites  Accuracy:  Information was compared to respective national dietary guidelines | 2 | No | Quality:  - Average information quality score was 3.45* (SD 0.67) (max. possible score 5)  - Maximum score was 4.2 and minimum score was 1.3  - 14 (93%*) websites scored above 3  - 1 (7%*) website scored below 3  Accuracy:  - Information contained in 14 (93%) of the websites was consistent with the respective national guidelines | Positive |
| Gholizadeh et al.^(37)^ 2017 | General | Iran | 51 | NR | All Persian websites related to nutrition and diet therapy were included.  No further information about how these websites were identified was reported. | ***Quality:  Tool used in a previous study adapted and seven quality components evaluated | NR | No | Quality:  - 34 (66.7%) websites had “mediocre” quality (60 contained accurate information)  - 17 (33.3%) had “acceptable” quality  - No websites had “poor” quality  - Average quality score for privately owned websites was 38.5 and organisational websites was 38 (max. possible score of 51)  Accuracy:  - 27 (52.9%) of websites had “mediocre” scores for accuracy | Negative |
| Lambert et al.^(78)^ 2017 | Renal diet | None | 254 | April 2015 – July 2015 | Google, Yahoo and Bing searches  A list of renal diet search terms was used  Results on the first 7 pages of results were screened | Quality:  DISCERN Instrument  Accuracy:  Evaluated by two dietitians and first author compared to relevant evidence-based guidelines. If information contained any inaccurate information, it was coded as inaccurate overall. | 3 | No | Quality:  - 126 (49.6%) considered “poor quality” (60 contained accurate information)  - 62 (24.4%) considered “fair quality” (61 contained accurate information)  - 66 (26%) considered “good quality” (65 contained accurate information)  Accuracy:  - 186 (73.2%) contained accurate information  - 68 (26.8%) contained inaccurate information  - The rate of accuracy was significantly higher for websites compared to YouTube videos (18.0%; P < 0.0001). | Neutral |
| Cardel et al.^(65)^ 2016 | Weight loss | US (in Spanish) | 66 | February 2016 | Google search  30 search queries in Spanish developed through a survey of consumers  First 5 non-sponsored links screened | Quality:  Criteria from a previous study used  Accuracy:  Evaluated against literature and 2015 Dietary Guidelines for Americans | 3 | Yes  Krippendorff's Alpha = 0.66 | Quality:  - Average quality score 0.8 ± 0.8 (max. possible score of 6)  - Average score for commercial sites 0.8 ± 0.7, news/media sites 0.8 ± 0.8, blog sites 0.5 ± 0.6 and unclassified sites 1.3 ± 1.1. Difference between quality of sites not significant (P = 0.27)  - 45% of sites provided reputable references, 68% provided hyperlinks  - 73% of sites provided date of last update  - 94% of sites made unsubstantiated claims  Accuracy:  - Average accuracy score of 1.5 ± 0.7 (max. possible score of 4)  - Average score for commercial sites 1.5 ± 0.6, news/media sites 1.3 ± 0.8, blog sites 1.0 ± 0.5 and unclassified sites 2.2 ± 0.5. Difference between accuracy of sites was significant (P = 0.0009) | Neutral |
| Cabrera-Hernández et al.^(64)^ 2015 | General | None | 567 | July 2013 | Terminology from the LID Dictionary of Metabolism and Nutrition was used. A random sample of 386 terms from the dictionary were searched in the Spanish and English versions of Wikipedia.  If a Wikipedia page was available for the term in the English and/or Spanish Wikipedia it was included. | Quality:  Quality variables were developed by the authors  Accuracy:  Accuracy of nutrition-related terms was compared to definitions from the LID Dictionary of Metabolism and Nutrition | NR | No | Quality:  Spanish Wikipedia:  - Average references 7.63 ± 1.34  - External links 1.64 ± 0.18  - 5 pages rated by users as outstanding, 2 good  English Wikipedia:  - Average references 27.81 ± 2.47  - External links 2.93 ± 0.21  - 9 pages rated by users as outstanding, 12 good  Accuracy:  - Spanish Wikipedia: 3 pages (1.15%) had no accurate information  - English Wikipedia: 2 pages (0.52%) had no accurate information | Neutral |
| Modave et al.^(81)^ 2014 | Weight loss | None | 103 | November 2012 | Google search  30 unique search terms used based on a survey of individuals interested in weight loss  Sponsored Google ads and the first 5 results were screened | Quality:  Two previously used assessment tools were adapted for this study and included criteria for authorship and design  Accuracy:  A scoring system for nutrition information was created based on current literature | 3 | No | Quality:  Authorship (max. possible score of 6)  - Average for all sites 0.82 ± 0.96  - Average for commercial sites 0.38 ± 0.80, news/media sites 0.65 ± 0.79, blogs 0.43 ± 0.79, medicine/university/government sites 2.07 ± 0.92 and unclassified sites 1.11 ± 0.78  - Medical or university sites were least likely to provide unsubstantiated claims  Design (max. possible score of 6)  - Average for all sites 3.62 ± 0.74  - Average for commercial sites 3.37 ± 0.89, news/media sites 3.48 ± 0.72, blogs 4.05 ± 0.23, medicine/university/government sites 4.27 ± 0.27 and unclassified sites 3.70 ± 0.63  - Design scores were significantly different between website types (P = 0.001)  Accuracy:  - Average accuracy score for nutrition for all sites 1.57 ± 0.99 (max. possible score of 4)  - Average for commercial sites 1.09 ± 1.05, news/media sites 1.52 ± 0.91, blogs 2.52 ± 0.57, medicine/university/government sites 1.86 ± 1.11, unclassified sites 2.07 ± 0.28  - Nutrition accuracy scores were significantly different between website types (P = 0.001) | Positive |
| Hirasawa et al.^(70)^ 2013 | General | None | 48 | December 2010 | Google, Yahoo and Bing searches  Search terms: "healthy diet"  First 100 results from each search were screened | Quality:  Criteria for quality assessment developed by authors  **Accuracy:  Information assessed against 6 sets of dietary guidelines: Dietary Guidelines for Americans, DASH diet, USDA My Pyramid, ADA Nutrition Recommendations and Interventions for Diabetes, AHA Diet and Lifestyle Recommendations for Cardiovascular Disease Risk Reduction and, Healthy People 2010 | 2 | No | Quality:  - 5 (10.4%) of sites provided references  - 26 (54.2%) provided date of last update  - 11 (22.9%) directed individuals with to a physician or dietician  - No significant difference between quality variables and website type  Accuracy:  - Mean matching score was 33.2% - 47.2% (percent of guidelines mentioned by websites accurately) | Neutral |
| Shahar et al.^(45)^ 2013 | Cancer | None | 100 | NR | Google search  Search terms: "nutrition + diet + cancer + prevention"  400 websites were screened | Quality:  Adapted quality criteria developed for a previous study  **Accuracy:  A scoring system was developed and information was compared to the WCRF/AICR cancer prevention guidelines | NR | No | Quality:  - 54% of the websites were categorised as “poor”, 45% “moderate” and 1% “satisfactory”  - 69% of sites provided references  - 86% of sites provided interactivity with users (e.g., forums)  - 41.7% included contact information  - 52% provided the author’s name, and of those, 82.7% included the author’s background  - 56% did not provide date of last update  - 12% received support from companies or public donations, and most had no advertisements  Accuracy:  - Due to the incompleteness of the content, 91% of sites had “poor” accuracy scores  - 10.3% included all 10 main recommendations for cancer prevention  - 2% did not include any WCRF/AICR recommendations  - Quality scores correlated significantly with accuracy scores (r = 0.250, p < 0.05) | Negative |
| McNally et al.^(79)^ 2012 | Coeliac disease | US | 98 | November 2010 | Google, Bing and Yahoo searches  Search terms: “celiac disease”  First 100 results screened | Quality:  Criteria developed based on other quality assessment tools (JAMA Benchmarks, HONCode and WebMedQual scale)  Accuracy:  Scoring system developed based on recommendations from the Warren Medical Research Center for Celiac Disease | 3 | Yes  Intraclass correlation coefficient  Quality:  r = 0.79, (95% CI 0.59-0.90)  Accuracy:  r = 0.75, (95% CI 0.52-0.88) | Quality:  - Quality scores ranged from 0.27 to 0.80 (max. possible score of 1.0)  - Average quality score was 0.52 ± 0.12  - Post hoc pairwise comparisons revealed that academic websites had significantly lower quality than commercial websites (P = .005)  Accuracy:  - Overall accuracy scores ranged from 0.62 to 1.00 (max. possible score of 1.0)  - Average accuracy score was 0.93 ± 0.07  - A minimum of 95% accurate information was obtained by 51 (52%) of websites  - Mean and median accuracy scores did not significantly differ by type of website | Neutral |
| Joshi et al.^(74)^ 2011 | Osteoporosis | None | 74 | 2009 | Google, Yahoo, Bing, AOL and Lycos searches  Search terms: "osteoporosis", "diet", "nutrition" and "bone loss".  First 20 websites for each search were screened | Quality:  DISCERN Instrument  **Accuracy:  Website content was measured on a scale and considered to be complete and accurate if the information included defining osteoporosis, its signs and symptoms, diagnosis, treatment options, and complications. No further information about how accuracy was determined was provided. | 2 | Yes  Ratings were significantly different between the two raters (P < 0.0001 (95% CI −1.74; −1.23) | Quality:  Average overall DISCERN scores (max. possible score of 5)  - Rater one: 3.08 ± 0.92  - Rater two: 1.59 ± 0.96  - Highest information quality scores were given to the .Org sites and the lowest to the .Gov sites  Accuracy:  - Average accuracy score for .Gov sites was 2.01 (max. possible score of 5)  - Average score for .Com sites was 2.27  - Average score for .Edu sites was 2.36  - Average score for 'other' sites was 2.36  - Average score for .Org sites was 2.91 | Neutral |
| Dornan et al.^(90)^ 2006 | Breastfeeding | US | 30 | June 2004 | Google, Yahoo and MSN searches  Search terms: "breastfeeding" "breast feeding"  First 5 pages for each search screened | Quality:  Health Information Technology Institute criteria  **Accuracy:  Information compared to AAP policy statement on breastfeeding (8 recommendations assessed) | NR | No | Quality:  - Authors or developers of the sites were identified in 21 (70%) of the 30 sites.  - 5 (16.7%) sites stated when the Web site was established, and 11 (36.7%) indicated when it was last updated  - 17 (56.7%) were useful for basic information about breastfeeding  - 19 (63.3%) reported research studies, expert opinion, or reputable organizations to support the information presented  - 20 (66.7%) had a purpose statement  - 18 (60%) contained quality links, and 12 (40%) contained no links or low-quality links  - 20 (66.7%) had easy to use search engines, 10 (33.3%) had none or poor-quality search engines  - 26 (86.7%) had a way for users to give feedback  - 6 (20%) had message boards or chat rooms  - 1 contained unsubstantiated claims  Accuracy:  - 7 (23.3%) of the 30 sites accurately mentioned all 8 AAP recommendations | Neutral |
| Hires et al.^(92)^ 2006 | General  (information published by Nutrition Consultants and Registered Dietitians) | US | 40 | October 2002 – November 2002 | Search engine NR  Search terms:  "dietitian", "dietitian consultant", or "nutrition consultant" | ***Quality:  Authors developed criteria, informed by JAMA Benchmarks.  Accuracy:  Five-point scale for accuracy assessment under quality criteria. No further information about what informed assessment. | 5 | No | Quality:  - Means for websites by dietitians ranked higher than nutrition consultants’ sites in 13 of 19 categories  - Dietitians achieved a mean average between 4.0 and 4.99 or "good" ranking in 6 categories compared to 4 for Nutrition Consultant sites  - Dietitian sites scored mean between 3.0 and 3.99 or "fair" ranking in 8 categories compared to 6 for Nutrition Consultant sites  - Dietitian sites scored a mean between 2.0 and 2.99 or "poor" ranking in 2 categories compared to 5 for Nutrition Consultant sites  - Dietitian sites scored a mean between 1.0 and 1.99 or "very poor" ranking in 2 categories compared with 3 for Nutrition Consultant sites  Accuracy:  - Dietitians’ average accuracy subscore 4.38 ± 0.40 (max. possible score of 5)  - Nutrition Consultants' average 3.66 ± 0.54  - Dietitian sites scored significantly higher for accuracy (P 0.0001) | Negative |
| Shaikh & Scott ^(46)^ 2005 | Breastfeeding | None | 40 | NR | Google, AOL and Yahoo searches  Search terms: "breastfeeding", "breast-feeding" and "breast feeding"  First two pages of results screened | Quality:  HONCode Principles  **Accuracy:  An evaluation tool was developed, and information was compared to AAP publications and contained four information categories related to breastfeeding | 2 | No | Quality:  - Average HONCode score was 1.5 ± 4.3 for all sites (max. possible score of 8, min. possible score of -8)  - Non-profit and professional organizations had the highest and commercial and “other” sites had the lowest ratings. Differences were not statistically significant.  - Noncompliance was greatest (50%) for complementarity  Accuracy:  - Average accuracy scores were 10.4 ± 7.9 for all sites (max. possible score of 24)  - 55.3% of the content items were not mentioned  - 42.4% of items were discussed accurately  - 2.3% of items were inaccurate  - Non-profit organizations had the highest scores, while academic institutions had the lowest. Low scores were usually because information was not included. | Negative |
| Sutherland et al.^(48)^ 2005 | General | US | 110 | NR | A general and a focused search conducted  General search: Google, Lycos, Alta Vista  Search terms: "+ nutrition + diet"  First 37 urls for each search engine were screened.  Focused search: 40 websites randomly selected from US Department of Health and Human Services directory to consumer health information  10 pages from each included website were evaluated | ***Quality:  A 27-item quality assessment tool was developed for this study.  Accuracy:  Accuracy was evaluated under the quality assessment tool. Information was compared to the Dietary Guidelines for Americans and Healthy People 2010. | 2 | Yes  ĸ = 0.76 | “Accuracy” and “currency” measures both include components of accuracy and quality.  Content "accuracy" scores:  - 2.63 ± 0.78 for government recommended sites (focused search)  - 1.84 ± 0.87 for other sites (general search)  - P <0.0001 (CI 0.496-1.084)  - Overall, .gov and .org Web sites provided more accurate content than ".com" sites  Content "currency" scores:  - 1.80 ± 0.48 for government recommended sites (focused search)  - 1.64 ± 0.56 for other sites (general search)  - Not significantly different | Neutral |
| England et al.^(91)^ 2004 | Coeliac disease | UK | 63 | September 2002 – October 2002 | MSN, Yahoo, Google searches  Search terms:  "Coeliac", "celiac" and "gluten intolerance"  All search results screened | Quality:  JAMA Benchmarks  Accuracy:  Information checklist for the management of coeliac disease was developed by the authors based on guidelines by the British Gastroenterology Society, the Manual of Dietetic Practice and the literature | 1 | No | Quality:  - Mean score 12.7 ± 4.7 (max. possible score of 27)  - No sites scored 27, 24 sites (38.1%) scored >13, 2 sites (3.2%) scored > 20  Accuracy:  - 15.9% of websites contained potentially harmful inaccuracies  Gluten-free diets (max. possible score of 14):  - Mean score of 6.7 ± 3.4  - 1 site (1.6%) scored 1, 12 sites (19.1%) scored <4, 28 sites (44.4%) scored >7 and 1 site (1.6%) scored 14  Nutrition & general management (max. possible score of 16):  - Mean score was 4.0 ± 3.2  - No sites scored 16, 7 sites (11.1%) scored 0, 1 site (1.6%) scored >12, 32 (50.8%) sites scored <4  - Accuracy and quality scores were compared with Pearson's correlation. No relationship observed (r 0.18, n 63) | Negative |

*Calculation for figure performed by ED from data extracted from study’s results section or supplementary material
**Assessment of accuracy encompassed measure of information comprehensiveness and evaluated if certain information was present

***Quality criteria included assessment of information accuracy

^Data provided by authors upon request

Abbreviations: AAP: American Academy of Paediatrics; ADA: American Diabetes Association; ADG: Australian Dietary Guidelines; AHA: American Heart Association; AGHE: Australian Guide to Healthy Eating; AICR: American Institute for Cancer Research; CI: Confidence Interval; DASH: Dietary Approaches to Stop Hypertension; EQIP: Ensuring Quality Information for Patients; HONCode: Health on the Net Code; HSE: Irish Health Service Executive; IPDAS: International Patient Decision Aid Standards; MARS: Mobile App Rating Scale; NHMRC: National Health and Medical Research Council; NR: Not reported; UK: United Kingdom; US: United States; USDA: United States Department of Agriculture; WCRF: World Cancer Research Fund
