# Supplementary material for: Quality and accuracy of online nutrition-related information: a systematic review of content analysis studies
Source: Public Health Nutr. 2023 May 4;26(7):1345–57. doi: 10.1017/S1368980023000873 (PMC10346027; doi:10.1017/S1368980023000873)
Supplement: Supplementary file 1 [file S1368980023000873sup.zip › S1368980023000873sup002.docx]

Supplementary Table 3: Study characteristics, results and risk of bias assessments of content analysis studies evaluating quality and/or accuracy of nutrition-related information on social media

| **Study, year** | **Nutrition-related topic** | **Social media platform** | **Country** | **Sample size** | **Data**  **collection period** | **Search & selection strategy** | **Evaluation method/tool(s) used** | **No. of raters** | **Inter-rater reliability assessed?** | **Key findings** | **Risk of bias assessment** |  |
| --- | --- | --- | --- | --- | --- | --- | --- | --- | --- | --- | --- | --- |
| **Quality** | | | | | | | | | | | | |
| Sabbagh et al.^(44)^ 2020 | Weight loss | Blogs | UK | 180 posts (from 9 blogs) | NR | Influencers were identified through marketing website ‘influence.co’, filtered by ‘United Kingdom’ and ‘nutrition  Google search using search terms: “nutrition”, “diet”, “physical activity”, “weight management”, “obesity”, “blog” and “influencer”  20 most recent relevant blog posts by UK based influencers included | Quality criteria developed for study based on systematic review results  Thirteen criteria were included, and scores reported as a percentage | NR | No | - Average quality score was 49%  - Maximum score was 85% and minimum 23%  - 6 (67%) influencers did not distinguish fact from opinion, providing no or inadequate references  - 2 (22%) influencers did not disclose advertising  - 5 (56%) did not provide a disclaimer and 4 (44%) did not include a privacy policy  - Authorship was clear for all blogs  - 2 (22%) influencers were adequately qualified to provide nutrition and weight loss advice  - Occupations were: 2 personal trainers, 1 medical doctor, 2 chefs, 1 registered associate nutritionist, 1 nutritional therapist and 2 unknown.  - The registered nutritionist had the highest quality/credibility score of 85%. | Neutral |  |
| Basch et al.^(35)^ 2016 | Supplements (multivitamins) | YouTube | None | 97 videos | NR | YouTube search  Search terms: "multivitamin supplement" and "vitamin"  100 most viewed videos screened | Content of videos was evaluated (no further details provided) | 2 | Yes  ĸ = 0.98 | - 80.4% mentioned benefits and 72.2% advocated for use of the supplement  - 84.5% did not mention risks associated with taking the supplement  - 45.5% referred to research  - 42.3% reported how much of a supplement to take  - 8.2% mentioned safety  - 42.9% (95% CI: 24.6%, 61.2%) of videos uploaded by television and internet sources mentioned risks  - 60.7% of videos uploaded by television and internet sources (95% CI: 42.6%, 78.8%) and 55.6%, by medical professionals (95% CI: 23.1%, 88.1%) referred to previous studies or research | Neutral |  |
| **Accuracy** | | | | | | | | | | | | |
| AlKhaja et al.^(60)^ 2018 | Supplements | WhatsApp | Kingdom of Bahrain | 4 messages | June 2016 – September 2017 | Convenience sample from messages received on WhatsApp during the study period that were related to dietary supplements | Information was compared to clinical evidence, FDA, European Medicines Agency, CDC and international treatment guidelines  Messages were classed as: “true”, “potentially misleading” or “false” | 2 | No | - *75% of claims/messages about the supplements were "potentially misleading"  - *25% of claims/messages were "true" | Negative |  |
| Koball et al.^(76)^ 2018 | Bariatric surgery and nutrition | Facebook | None | 169 posts | May 2016 | Facebook searched for bariatric surgery groups  A random selection of posts examined from consenting groups with the highest number of members | Registered dietitians coded the information based on clinical expertise and ASMBS expert nutrition guidelines, Obesity Society, ASMBS, and the AACE clinical practice guidelines | 3 | No | - 11 (7%) of posts were inaccurate  - 38 (22%) of posts contained both accurate and inaccurate information  - 41 (24%) of posts contained information that was too ambiguous to evaluate  - 79 (47%) of posts contained accurate information | Positive |  |
| Kiedrowski et al.^(42)^ 2017 | Coeliac disease | YouTube | Poland | 91 videos | NR | YouTube search  Search terms: "celiakia" (celiac disease), and "choroba trzewna" (Polish synonym for celiac disease)  Number of results screened NR | Information assessed by 3 doctors with relevant expertise and 1 dietitian. Information classed as “credible” or “non-credible” based on the assessors’ knowledge | 4 | No | - 87 (95.6%) videos that contained culinary information and recipes for celiac disease were classed as credible | Negative |  |
| Alnemer et al.^(97)^ 2015 | General | Twitter | None (written in Arabic) | 101 tweets | April 2015 | Twitter search  Search terms related to healthy diets and nutrition in the Arabic language were used  Dietitian accounts were identified as those whose bio provided a link to their clinic or hospital website | Information classed as false, true with weak, true with moderate, or true with strong evidence based on expert opinion (reviewers collaborated with experts in specific fields if needed). | 3 | Yes  Formula for agreement calculation:  (true/[true + false])  Expert 1: 0.57  Expert 2: 0.78  Expert 3: 0.22 | - 59 (58.4%) of dietitian's tweets were classified as false  - 42 (41.6%) of dietitian's tweets were classified as true | Negative |  |
| **Quality & accuracy** | | | | | | | | | | | | |
| Batar et al.^(98)^ 2020 | Bariatric surgery and nutrition | YouTube | None | 114 videos | February 2020 | YouTube was searched using terms: "after bariatric surgery diet", "weight loss surgery postop diet"  First 100 videos for each keyword used with YouTube filtering for "most relevant" | Quality:  - DISCERN Instrument  - JAMA Benchmarks  - GQS  - Usefulness score  Accuracy:  Scoring system developed based on guidelines by the American Society for Metabolic and Bariatric Surgery | 2 | No | Quality:  - Average DISCERN score: 31.58 ± 10.02 (max. possible score 80)  - Average JAMA score: 2.25 ± 0.97 (max. possible score 4)  - Average GQS score: 2.35 ± 0.98 (max. possible score 5)  - Average Usefulness score: 3.1 ± 1.9 (max. possible score 10)  - The DISCERN, JAMA, GQS, and Usefulness scores were significantly higher in physician- or dietician-based videos than in patient-based videos (P < 0.001).  Accuracy:  - Average accuracy score: 3.59 ± 1.82 (max. possible score 10)  - Accuracy scores did not significantly differ between video sources (P >0.05) | Negative |  |
| Mete et al.^(80)^ 2019 | General | Blogs | Australia | 76 posts (from 5 blogs) | December 2018 – March 2019 | Google, Bing and Yahoo searches  Search terms: “Australian Healthy Eating Blogs” and “Top 100 Australian Healthy Eating Blogs”  First page of results screened | **Quality:  HRWEF and SAM were adapted to create a coding scheme to guide analysis  Accuracy:  Information was compared to the ADG | 2 | No | Quality:  - 97% of posts explicitly stated their purpose  - 100% used a conversational writing style  - 100% used common words, explained jargon and used imagery  - 100% adhered to layout criteria  - 64% provided procedural knowledge, 17% declarative knowledge (with at least 40% procedural knowledge as well), 11% declarative knowledge only and 8% not applicable  Accuracy:  - 43% of posts explicitly adhered to the ADG  - 17% of posts somewhat adhered  - 7% of posts did not adhere  - 33% of posts contained information that was not applicable | Neutral |  |
| Toth et al.^(89)^ 2019 | Detox diets | Blogs | Canada | 10 blogs  (5 by nutritionists and 5 by dietitians) | November 2017 | Google searches  Search terms: "detox diet nutritionist Ontario blog" and "detox diet dietitian Ontario blog" | Quality:  Criteria developed by authors.  Accuracy:  Compared to findings from relevant systematic review | 2 | Yes  96% agreement | Quality:  - 80% of nutritionists and 0% of dietitians were selling a service related to detox diets  - 40% of the dietitians used references, including peer-reviewed journal articles  - References mentioned by nutritionists were not from peer-reviewed journals  - 20% of nutritionists and 100% of dietitians were university educated  Accuracy:  - 11% of nutritionists' statements were consistent with current evidence  - 96% of dietitians' statements were consistent with current evidence | Positive |  |
| Reddy et al.^(84)^ 2018 | Food allergies | YouTube | None | 300 videos | June 2016 – July 2016 | YouTube search  Search terms: "food allergy" "food allergies"  300 most frequently viewed videos that met inclusion criteria were included | Quality:  GQS  Accuracy:  A tool was developed for this study based on authoritative guidelines, literature, and expert knowledge | 8 | Yes | Quality:  - Average GQS of 2.4 (max. possible score of 5)  Accuracy:  - 26.3% of videos contained misleading information.  - Average accuracy score was 4.05 (max. possible score of 34)  - Average score for professional societies was higher than all other sources (all P < .001) | Neutral |  |
| Lambert et al.^(78)^ 2017 | Renal diet | YouTube | None | 161 videos | April 2015 – July 2015 | YouTube searches  A list of renal diet search terms was used  Results on the first 7 pages of results were screened | Quality:  DISCERN Instrument  Accuracy:  Evaluated by two dietitians and first author compared to relevant evidence-based guidelines. If information contained any inaccurate information, it was coded as inaccurate overall. | 3 | No | Quality:  - 94 (58.4%) considered “poor quality” (6 contained accurate information)  - 48 (29.8%) considered “fair quality” (10 contained accurate information)  - 19 (11.8%) of YouTube videos were considered “good quality” (13 contained accurate information)  Accuracy:  - 29 (18%) contained accurate information  - 132 (82%) contained inaccurate information | Neutral |  |
| Rhoades et al.^(85)^ 2010 | Food safety | YouTube | None | 76 videos | “Mid-summer”  2007 | YouTube search  Search terms: "food safety"  81 results returned and all were screened | **Quality:  Criteria developed by authors  Accuracy:  Accuracy of information assessed based on professional knowledge/opinion | 2 | Yes  Inter-coder Holsti’s reliability = 90.7% | Quality:  - Average score was 3.7 ± 1.1 (max. possible score of 5)  - 28 (36.8%) videos cited or showed a source.  Accuracy:  - 1 video (3.1%) contained researcher-identified falsehoods. | Negative |  |

*Calculation for figure performed by ED based on results or supplementary material

**Quality criteria included assessment of information accuracy

Abbreviations: AACE: American Association of Clinical Endocrinologists; ADG: Australian Dietary Guidelines; ASMBS: American Society for Metabolic and Bariatric Surgery; CDC: Centre for Disease Control; FDA: Federal Drug Administration; GQS: Global Quality Score; HRWEF: Health-Related Website Evaluation Form; JAMA: Journal of the American Medical Association; NR: not reported; SAM: Suitability Assessment of Material; UK: United Kingdom
